# “An Adventure That Went Wrong”: Reasons Given by Convicted Perpetrators of Multiple Perpetrator Sexual Offending for Their Involvement in the Offense

**DOI:** 10.1007/s10508-017-1011-8

**Published:** 2017-08-07

**Authors:** Teresa da Silva, Jessica Woodhams, Leigh Harkins

**Affiliations:** 1 School of Psychological, Social and Behavioural Sciences, University of Coventry, Coventry, CV1 5FB UK; 2 School of Psychology, University of Birmingham, Birmingham, B15 2TT UK; 3 Faculty of Social Science and Humanities, University of Ontario Institute of Technology, Oshawa, ON L1H 7K4 Canada

**Keywords:** Multiple perpetrator rape, Multiple perpetrator sexual offending, Group rape, Group sex offending, Sex offenders

## Abstract

There is little empirical research examining the reasons behind multiple perpetrator sexual offending. A limited number of studies provide reasons for offending offered by perpetrators of this type of sexual violence, but only one published study exists where these perpetrators were interviewed regarding their offense. The Multi-Factorial Model of Multiple Perpetrator Sexual Offending (MPSO) proposed that various factors (individual, sociocultural, and situational) play a role in this type of sexual assault, noting in particular the importance of group dynamics and processes. In the current study, 25 convicted perpetrators of multiple perpetrator sexual offending housed in educational centers and prisons in Portugal were interviewed about their involvement and reasons for participating in the offense. The findings suggested that group processes and dynamics play an important part in this type of sexual offending. Furthermore, the results provided some evidence to support the factors proposed by the Multi-Factorial Model of MPSO. These findings have implications for prevention and treatment programs and for the assessment of offenders.

## Introduction

Multiple perpetrator sexual offending (MPSO)^1^ is an international phenomenon that has been present throughout history and which manifests in various settings, including street gangs, college fraternities, sports teams, in prison, and during war and civil unrest (da Silva, Harkins, & Woodhams, 2013). In the U.S., it is estimated that between 10 and 33% of sexual assaults are committed by multiple perpetrators (Franklin, 2004). Similar figures have been reported in countries such as South Africa (9–27%; Jewkes, Sikweyiya, Morrell, & Dunkle, 2009), Australia (23%; Australian Bureau of Statistics, 2004), and the UK (11–19%; Curran & Millie, 2003; Kelly, Lovett, & Regan, 2005; Wright & West, 1981).

The majority of research conducted on MPSO has utilized archival data, such as police reports and victim statements (Amir, 1971; Chambers, Horvath, & Kelly, 2010; da Silva, Woodhams, & Harkins, 2014; Horvath & Kelly, 2009; Woodhams, 2008; Wright & West, 1981), court files (Bijleveld & Hendriks, 2003; Bijleveld, Weerman, Looije, & Hendriks, 2007), and law reports (Hauffe & Porter, 2009; Porter & Alison 2006). These studies have been useful in providing information regarding the characteristics of offenders, victims, and offenses. They do not provide information about the reasons and reported explanations for the assault, however, which are important when trying to intervene therapeutically with the offenders. This information can be obtained from the perpetrators of MPSO directly although this methodology has rarely been adopted by researchers. This paper presents the findings of a study in which perpetrators of MPSO were interviewed to explore their role in the MPSO and the reasons and explanations offered for their involvement. The findings are compared to the factors proposed in Harkins and Dixon’s (2010) Multi-Factorial Model of MPSO.

### Theories of Sexual Offending and MPSO

Existing theories of MPSO propose a variety of contributory factors that include individual and sociocultural factors, and group processes (Amir, 1971; Brownmiller, 1975; Groth & Birnbaum, 1979; Harkins & Dixon, 2010, 2013; Sanday, 2007). The most recent and comprehensive theory, which draws together the factors proposed by earlier theories, is the Multi-Factorial Model of MPSO developed by Harkins and Dixon (2010, 2013).^2^

This model proposes that various factors (individual, sociocultural, and situational), and the interactions between them can explain the occurrence of MPSO (Harkins & Dixon, 2010, 2013). Individual factors that contribute to MPSO include personality traits (e.g., leadership), developmental factors, and paraphilic sexual interests. Sociocultural factors, such as cultural norms, myths, beliefs, and values about women, sexuality, and violence are also thought to play a role. In particular, rape culture, rape myths, patriarchy, and negative or stereotypical attitudes and beliefs about women are conducive to MPSO. Situational factors are proposed to facilitate the occurrence of MPSO by helping overcome any inhibiting factors, or by acting as a trigger. This would include specific contexts, such as fraternities and wars, where exaggerated sexuality is common or hostile masculinity is acceptable.

The model explains that individual, sociocultural, and situational factors interact in diverse ways further contributing to the likelihood of a MPSO occurring (Harkins & Dixon, 2013). Three possible interactions are: internalization of sociocultural factors (between the individual and the sociocultural context); group processes (between the individual and situational factors), and subcultural context (the situational context and sociocultural factors). Harkins and Dixon (2013) suggested that the internalization of sociocultural factors is associated with the degree to which a person internalizes sociocultural norms and how these influence their beliefs and cognitions. They emphasized the importance of group processes in the perpetration of MPSO, including social comparison, social dominance, conformity, obedience to authority, social corroboration, deindividuation, and groupthink. Lastly, the interaction between specific situational contexts and broader sociocultural factors produces the subcultural context. This means that given a particular type of situation, certain cultural practices could lead men to commit a sexual offense as a group.

There are also numerous theories of general sexual offending which range from single- to multifactor models (Ward, Polascheck, & Beech, 2006) that could help explain MPSO. Of these, the most comprehensive theory that attempts to incorporate previous theories of sexual offending is the Integrated Theory of Sexual Offending (ITSO) (Ward & Beech, 2006). This theory proposes that sexual abuse occurs because of the interaction of several causal factors. These include biological factors (influenced by genetic inheritance and brain development), ecological niche factors (social and cultural environment, personal circumstances, and physical environment), and neuropsychological factors (e.g., motivation/emotion, perception and memory, and action selection and control). The ITSO includes all of the factors proposed by the Multi-Factorial Model of MPSO (individual, sociocultural, and situational) and effectively explains individual sexual offending; however, it does not specifically include the group processes that are argued to be so important in explaining MPSO.

In contrast with the sexual offending literature, the group’s influence is given explicit consideration in theories of general co-offending. Within the literature on co-offending, it is possible to identify three basic perspectives (Weerman, 2003): the group influence, social selection, and the instrumental perspective. The group influence perspective proposes that co-offending is explained by group processes (e.g., social learning, acquisition of delinquent definitions, and group pressure) which lead to social rewards (Akers, 1973; Matza, 1964). On the other hand, the social selection perspective argues that criminal groups form because offenders that share similar characteristics select each other (Hirschi, 1969; Kornhauser, 1978). According to the instrumental perspective, co-offending is the result of a decision making process where the offenders weigh the advantages and disadvantages of lone versus co-offending and select co-offending because it is easier, less risky, and/or more profitable (Letkemann, 1973; Walsh, 1986).

Theories of general co-offending have been criticized for being underdeveloped (Weerman, 2003), and there have been calls for more empirical research to assess their relevance (McGloin & Nguyen, 2012). In terms of their value in explaining MPSO, they are not as comprehensive as the ITSO (Ward & Beech, 2006) or the Multi-Factorial Model of MPSO (Harkins & Dixon, 2010) as they neglect to consider individual factors.

### Past Studies with Perpetrators of MPSO

Only a limited number of studies have asked perpetrators of MPSO about their reasons for participating in the offense, and these questions were not the main focus of the research studies (Etgar & Ganot-Prager, 2009; Hooing, Jonker, & van Berlo, 2010; Jewkes et al., 2006; Jewkes, Sikweyiya, Morrell, & Dunkle, 2011; Scully & Marolla, 1985). Etgar and Ganot-Prager (2009) examined the advantages of including adolescents who participated together in the same MPSO in the same therapeutic group and reported what the adolescents said in group therapy about their involvement in the assault. The young offenders often reported a need for social acceptance or feelings of social pressure as reasons for their involvement in MPSO. Illustrative quotes included statements about wanting to belong and become one of the group, and fears of rejection if they did not participate.

The characteristics of juvenile sex offenders (including perpetrators of MPSO, *n* = 142) in a mandatory sex education program were analyzed by Hooing et al. (2010). Explanations for and feelings about the crime that were described by the young offenders at the beginning of the program were examined. Multiple perpetrator sexual offenders had more negative attitudes toward girls compared with lone sex offenders who had assaulted a peer. Additionally, 50% (*n* = 72) of the multiple perpetrator sexual offenders stated that an important motive for offending was group pressure or group dynamics. For these offenders, non-sexual reasons for participating in this type of offense, such as those related to sociability and social dominance, were more prevalent than sexual motives, such as sexual arousal. In fact, only 13% (*n* = 18) stated that sexual arousal was a reason for the offense.

In South Africa, survey studies were conducted with men from the community (*n* = 1686) where, among other questions related to health issues, male adolescents and adults were asked about the perpetration of rape, including MPSO (Jewkes et al., 2006, 2011). Among these men, 9% (*n* = 149) had committed a multiple perpetrator sexual offense. The reasons that were given by the men for their involvement in MPSO included: sexual entitlement, boredom, fun, alcohol consumption, peer pressure, and a desire (motivated by anger) to punish girls or women who did not conform to stereotypical gender norms (e.g., the women and girls were considered promiscuous, or drank alcohol and smoked, or were lesbians).

Scully and Marolla (1985) interviewed convicted rapists (*n* = 114), including multiple perpetrator rapists, and asked a number of open-ended questions on the offenders’ own perceptions of their crimes. The most common reasons given by multiple perpetrator rapists for participating in an assault were related to recreation and adventure. Male camaraderie was also highlighted as important, which was achieved by participating together in a dangerous and illicit activity.

The only published study that focused exclusively on interviews carried out with perpetrators of MPSO was conducted by Blanchard (1959). In order to further understand the group process in MPSO, Blanchard interviewed seven teenage boys who had been involved in one of two different multiple perpetrator sexual offenses (three belonged to one group and four to the other group). At that time, psychologists based their explanations for this type of sexual violence on psychodynamic theory (which considered the relevance of homosexual factors in MPSO). Blanchard carried out psychological tests, including the Rorschach, which were administered individually and then to the group. Blanchard claimed that some of the results suggested the existence of homosexual factors: The sexual feelings identified in one of the rapes were to a great extent between the perpetrators instead of between any of the perpetrators and the victim. In the final conclusions, Blanchard identified a clear leader in both of the cases and stated that they were sexually stimulated by the presence of the group. However, he thought that in one of the cases, the sexual feelings that were stimulated did not appear to be homosexual. Instead, the leader was thought to be defending himself against the fear of being weak or not masculine enough. Blanchard highlighted the importance of the leader and argued that a central factor in a group rape is the degree to which the leader is able to direct the attention of the other members of the group to sexual issues. Additionally, he noted that the group dynamics operating between the leaders and the rest of the group members during the group evaluations were similar to the dynamics present during the actual assault.

In conclusion, most of these studies reported that many of the reasons given by participants for taking part in MPSO were non-sexual. Furthermore, they suggest that group processes and dynamics play an important role in MPSO.

### Rationale

One of the most effective ways of gathering information regarding reasons for offending and the role of group dynamics is from the perpetrators of MPSO themselves. As noted above, very few studies have adopted this approach and the main focus of those studies was not the offenders’ account of their reasons for participating in the offense. There is only one published study (Blanchard, 1959) where the focus was exclusively on interviewing perpetrators of MPSO. However, this study had a very limited sample size (of teenagers only), is more than 50 years old, and focused mainly on examining if there were homosexual factors present in MPSO.

In an effort to address this gap in the MPSO research, the authors of the current study sought to interview convicted perpetrators of MPSO about their involvement, experiences and reasons for participating in the offense. The current study addressed the following research question: What reasons do convicted perpetrators of MPSO give for their involvement in the offense? It is important to address this research question because it is pertinent for prevention, assessment, and treatment purposes. For example, if empirical studies are able to demonstrate that group processes are a central part of this type of sexual offending (as is proposed by theories of MPSO), then these would be a clear target for prevention and treatment efforts, and are relevant to the assessment of offenders. Furthermore, there may be other factors unique to MPSO that need to be identified and taken into account. Since this is a qualitative study, formal hypotheses were not formulated a priori to avoid any potential bias in interpreting the results. However, based on the literature reviewed, it was expected that at least some of the offenders would cite group processes as related to their involvement in MPSO.

## Method

### Participants

A total of 25 offenders convicted of MPSO agreed to participate in the study, which is noted as an acceptable size for a qualitative study employing thematic analysis (Guest, Bunce, & Johnson, 2006). As can be seen in Table 1, the offenders ranged in age from 13 to 45 years (*M* = 19.3, *SD* = 8.5), although the majority (72.0%, *n* = 18) were juveniles aged from 13 to 17 years. Approximately half (52.0%, *n* = 13) were of African ethnicity, followed by White (36.0%, *n* = 9), Romany (8.0%, *n* = 2), and Mixed Race (4.0%, *n* = 1). In terms of education, their years of schooling ranged from 0 to 8 years (*M* = 5.4, *SD* = 1.6). More than half (56.5%, *n* = 13) were living with parent/s or were students (54.2%, *n* = 13) at the time of the offense.

**Table 1:** Participants’ characteristics

Participant	Age at the time of the offense	Ethnicity	Years of schooling at the time of the offense	Living with	Employment
P1	14	White	4	Parents	Student
P2	15	African	6	Single parent	Student
P3	16	African	–	–	–
P4	13	Romany	6	Parents	Student
P5	14	White	6	Relatives	Student
P6	15	White	5	Alone	Not in school or employment
P7	14	White	5	Parents	Student
P8	14	White	5	Parents	Student
P9	15	African	7	In an institution	Not in school or employment
P10	13	White	5	Parents	Student
P11	17	African	7	Parents	Student
P12	17	African	6	Parents	Student
P13	17	Mixed race	4	Single parent	Student
P14	16	African	6	On the streets (homeless)	Not in school or employment
P15	13	White	5	In an institution	Student
P16	15	African	6	Parents	Student
P17	17	African	8	Single parent	Not in school or employment
P18	45	African	6	Room mate	Employed
P19	29	White	4	Spouse	Employed
P20	20	African	6	–	Unemployed
P21	17	Romany	6	Relatives	Student
P22	25	African	4	Room mate	Employed
P23	25	African	8	Relatives	Employed
P24	43	White	0	Parents	Employed
P25	23	African	6	Parents	Unemployed

#### Offense and Victims

The interviews related to 21 different offenses. As can be seen in Table 2, for four of these offenses, two different offenders who had participated in the same offense were interviewed. The number of offenders present in the offenses ranged from 2 to 8 (*M* = 3.5, *SD* = 1.7). In 16 cases (76.2%) the victims were female; the remaining five (23.8%) were male. In approximately two-thirds of the offenses (61.9%, *n* = 13) the victims were known to at least one of the offenders. The majority of the offenses occurred while the offenders were socializing (66.7%, *n* = 14). In four cases (19.1%) they occurred during a robbery. In cases involving couples, the victim was moved to a different location from their partner for the offense. The remaining three cases (14.3%) involved male school-age victims and two occurred at school in changing rooms. The last one occurred outside of school and the offenders claimed that their intention was to punish the victim.

**Table 2:** Characteristics of the offenses and victims

Offense	Participants	Number of offenders	Circumstance/Planned	Victims		
				Number/age	Gender	Relationship
1	P1	5	Social/unplanned	1/12	Female	Known
2	P2, P3	8	Social/unplanned	1/13	Female	Known
3	P4, P5	4	Social/unplanned	1/13	Female	Known
4	P6	3	Social/unplanned	1/22	Male	Known
5	P7, P8	3	Punishment/unplanned	1/12	Male	Known
6	P9	3	Social/planned	1/13	Female	Known
7	P10	2	School/unplanned	1/13	Male	Known
8	P11, P12	5	Social/unplanned	1/16	Female	Known
9	P13	5	Social/planned	1/16	Female	Known
10	P14	3	Social/unplanned	1/16	Female	Known
11	P15	3	School/unplanned	1/10	Male	Known
12	P16	3	Social/unplanned	1/23	Female	Known
13	P17	2	Robbery/unplanned	1/adult	Female	Stranger
14	P18	2	Social/unplanned	1/adult	Female	Stranger
15	P19	3	Robbery/unplanned	Couple/adult^a^	Female	Stranger
16	P20	2	Robbery/unplanned	Couple/adult^a^	Female	Stranger
17	P21	3	Robbery/unplanned	Couple/adults^a^	Female	Stranger
18	P22	2	Social/unplanned	1/adult	Female	Stranger
19	P23	2	Social/unplanned	1/adult	Female	Stranger
20	P24	2	Social/unplanned	1/10	Male	Known
21	P25	2	Social/unplanned	2/adults	Female	Stranger

^a^Only the female was sexually assaulted

### Procedure

A research proposal was sent to the Portuguese Parole and Prison Services (Direção-Geral de Reinserção e Serviços Prisionais—DGRSP) requesting to interview offenders convicted of MPSO and access to their case files. The files included detailed court accounts of their offenses and the facts that were proved in court. The case files were read by the first author before the interview and the offenders were informed of this. The research proposal was granted full ethical approval by the Science, Technology, Engineering and Mathematics Ethical Review Committee at the University of Birmingham, UK. It was also approved by the DGRSP. The first author, who is fluent in Portuguese, was permitted access to five Educational Centers (where young offenders under the age of 16 are held) and four prisons (with offenders from age 16 upward). Offenders convicted of MPSO were approached individually by the first author who provided information about the study, including an information sheet. The offenders who agreed to participate signed a consent form.

The interviews were semi-structured using an interview schedule that consisted of open-ended questions related to what happened before, during, and after the offense and with prompts to elicit more detailed responses (e.g., Could you explain how the offense occurred? Could you explain what happened during the assault? What was your role? How did the offense end?). It should be noted that the interview schedule was not structured around assessing factors from any particular theories, such as the Multi-Factorial Model of MPSO (Harkins & Dixon, 2010). The interviews of 14 participants were audio-recorded with their permission. However, 11 participants did not want their interviews to be audio-recorded and instead, hand-written notes were made by the interviewer. Shorthand was used which facilitated the note taking and only the quotes that were verbatim are included in the paper. The interviews were conducted individually in a quiet room or office in the educational centers and prisons where privacy was guaranteed. They lasted between 20 min and 1 h. The shorter interviews were those conducted with the very young offenders (13–15 years) who struggled to talk about their reasons for being involved in the offense. The audio-recorded interviews were transcribed verbatim by the first author and then translated into English. The majority of the participants’ language skills were poor, and Portuguese was not the first language of some of the African participants. When translating the verbatim transcripts, the first author did not correct the poorly constructed sentences or the grammatical errors as the authors considered that it was important to have a true translation of the transcripts. The recordings were deleted after the transcripts were made. Any identifying information was omitted from both the transcripts and the hand-written interviews.

### Analysis

The study design was qualitative, and thematic analysis was used to analyze the interviews. The guidelines for conducting thematic analysis recommended by Braun and Clarke (2006, 2013) and Guest, MacQueen, and Namey (2012) were followed. An inductive “bottom up” analysis was conducted which was data-driven (Patton, 1990). The first author familiarized herself with the data while transcribing and translating the interviews. The translated transcripts were imported into NVivo10, a computer software package that facilitates the organization and analysis of qualitative data. The whole data set was read and re-read, and first ideas were noted. Next, initial coding was conducted in a systematic form across the entire data set. This was achieved by identifying interesting features of the data that were linked to corresponding codes or sub-codes. As new features were identified, additional codes were generated. When reoccurring aspects of the data were identified, these were linked to existing codes in the coding scheme. After all the data were coded, these codes and sub-codes were sorted and collated into potential themes. Thematic maps were employed to facilitate the sorting of codes and sub-codes into themes as they enabled the visualization of relationships. The initial themes were then reviewed and refined at the level of the coded extracts and in relation to the whole data set. Lastly each theme was further refined, defined and named. An iterative approach was utilized throughout the analysis where codes, sub-codes, themes and sub-themes were constantly re-examined, and revised when appropriate. For the purpose of this article, only the themes related to the research question of this study are presented (i.e., what reasons do convicted perpetrators of MPSO give for their involvement in the offense?).

## Results

Six themes were identified that related to reasons given by the participants for being involved in a MPSO: (1) started as something else, (2) influence of others (direct or indirect), (3) lack of insight, (4) victim blaming, (5) influence of alcohol and or drugs, and (6) normalized sexual violence (see Table 3). In most cases, not just one reason was given and it was common for the participants to consider various factors as having played a role in the offense. The interviews were compared to the court accounts in the offenders’ case files and it was found that the majority were similar to the court accounts, although some minimization was evident in several interviews.

**Table 3:** Themes and corresponding factors

Themes	Participants	Multi-factorial MPSO
Started as something else	92% (*n* = 23)	Combination of individual, sociocultural, and situational factors
Influence of others	48% (*n* = 12)	Group processes (social comparison and conformity)
Lack of insight	24% (*n* = 6)	Group processes (deindividuation)
Victim blaming	48% (*n* = 12)	Sociocultural factors
Influence of alcohol and/or drugs	24% (*n* = 6)	Situational factors
Normalized sexual violence	8% (*n* = 2)	Subcultural context

This table shows how the themes identified in the current study map on to the factors proposed by Harkins and Dixon’s (2010, 2013) Multi-factorial MPSO

### Started as Something Else

Most of the participants denied that they had planned to sexually assault the victims beforehand. Only two (8.0%) of the 25 participants admitted that the group had planned earlier to have sex with the victim. The rest of them (*n* = 23, 92.0%) stated that the offense had started out as something else, such as a game or joke, physical bullying, or a robbery:

P10: I was having a swimming lesson with those two colleagues and that started off as a joke (pause) and (pause) and I had no intentions of rape or anything. At that time, I (pause) didn’t know the consequences it could bring and (pause) so we started joking around and all of that but not (pause) not, it wasn’t intentional.

P8: We didn’t plan the sexual thing but we planned to beat him because he had made a complaint.

P20: Yeah, we left, left with the purpose of (pause) of going to rob and and (pause) we went (pause) to (pause) and when I realized what was happening (pause) pfff (pause) it had already happened, I don’t know.

Even in one of the cases where the participant admitted that they had planned among themselves to have sex with the victim, he stated that they had not discussed using force as they thought that she would be willing. He described a situation that started off as having fun with his friends and expecting that the victim would want to have sex with all of them because she was known to have participated in similar situations in the past. It all changed when the victim said that she was only willing to have sex with one of them.

P9…The three of us were already expecting that there was going to be sex between the four of us, no there were five, one walked away. We were already expecting but we weren’t also expecting that she wouldn’t, wouldn’t want to.

P9: …I didn’t intend to want to force, to want to force her. So this for me, I considered this an adventure that went wrong.

The participants were not able to clearly explain why the situation escalated into a sexual assault. A few pointed to factors related to loss of control, adrenaline, and an impulse, but as can be seen in the quotes below they also considered other factors such as influence of others or being drunk. It is possible that a combination of factors was present and played a role in the offenses.

P8: We didn’t control ourselves (pause) I don’t know.

P19: …I don’t know how to explain why I did it, if it was adrenaline or if I let myself be led.

P22: …I don’t know if it was an impulse or of being drunk.

### Influence of Others

Not surprisingly, since MPSO is an offense carried out in the presence of other people almost half (*n* = 12, 48.0%) of the participants spoke about the influence of others. This influence was either direct, where the participants had been ordered, told, or invited to participate in the offense by a co-offender, or indirect, where they were not directly ordered to participate but did so because the others were present or actively involved. Directly telling or ordering a co-offender to participate was only evident in a few cases. In some of these cases it took the form of a direct order (the quotes below correspond to what was in the court accounts in the offenders’ case files):

P7: It was at that time, one of them ordered (pause) he turned to the victim and ordered him to turn around (pause)

Interviewer: Yes and then?

P7: I was ordered to go first.

P15: I ordered him. I said like this: “Do that to him” (pause) and he did it.

In other cases, it occurred not as a direct order but at the insistence of a co-offender that he should take part in the offense:

P21: So I got there, the other one was doing it, that’s it, get there be faced with that, then they start to influence: “Oh come, come, take, go on, go on” and in that situation, it isn’t, it isn’t, I don’t know, it is things that (pause) the influence is such that you are so into that situation that you go.

This insistence also included taunting and making the co-offender look bad if he did not participate:

P18: We were all drunk and he then didn’t give up, he pushed me, pressured me “If you don’t go, you are a coward” and I ended up by accepting his invitation.

When participants referred to the indirect influence of others, they stated that the co-offenders had not told them to participate, but that they chose to do so themselves. This happened in some cases simply because they were seeing the others participate and either felt aroused or decided that they also wanted to be involved:

Interviewer: Was there someone who said to do that?

P3: No, I think it was because a person seeing someone having relations also becomes motivated.

Not wanting to look bad in front of the co-offenders and participating to avoid being rejected was also mentioned:

P9: Because I was, I was with (pause) how shall I explain (pause) because I didn’t (pause) want to appear weak, I didn’t want (pause) to, to have hassles. Not to be rejected by them. It was more for that and since I was there in the middle (pause) I also tried to go.

### Lack of Insight

Almost one quarter (*n* = 6, 24.0%) of participants described a lack of insight into their thoughts and feelings at the time that they participated in the offense. They had difficulty describing the assault or parts of the assault. This difficulty did not seem to be just related to the fact that it is a sensitive and difficult topic to talk about; they described the offense as being confusing or happening very quickly:

P13: I don’t know how to explain very well (pause) hmmm (pause) it was all confusing (pause) it was all a bit confusing (pause) hmmm.

P20: I don’t know (pause) pfff (pause) man that (pause) I don’t know really that was kind of (pause) pfff (pause) something very fast really (pause).

Furthermore, they were unable to explain why they took part in the assault or what might have influenced their behavior at the time.

P22:…even now I ask myself, what came over me I don’t know, I don’t know what came over me, a thing (pause) man a person doesn’t have an explanation.

P20:… I don’t know what crossed through my mind to do a thing like that, until today I also can’t think.

### Victim Blaming

As in lone sexual offending, it was also found that almost half (*n* = 12; 48.0%) of the offenders blamed the victim for the offense. This was done to differing degrees, which ranged from attributing all the blame to the victim to insinuating that the victim held some responsibility. A few participants directly stated that it was the victim’s fault because she/he had wanted to participate or came up with the idea:

P1: No, my crime was because she wanted to. She said that she would do that if we let her into the group, and my colleague said “Oh yes? Come on then”

Other participants did not attribute all the blame to the victim, but they did suggest that the victim had wanted to participate and then changed her/his mind later on:

P6: …but that guy that did this, he also did it because he wanted to. He then afterwards (pause) we started, started talking and making fun. So he did something like that and then went to complain to the police.

Additionally, the victim’s behavior at the time of the offense was also seen by some of the participants as contributing to the offense. One of the participants recalled how the victim had said that she only wanted to have sex with one of the members of the group but that she talked about her feelings for the other members of the group and that this led to some confusion:

P9: And also the conversation she was having because she just wanted to have with one, but then she would also say “Oh I like you a little bit, I used to like you more, I like him a little bit” and I don’t know what. We all stayed with that thing in our head. In the end she just wanted to have it with that one, with that one. It was (pause) it was a bit confusing.

Finally, some participants spoke about the victim’s past behavior and her/his reputation of having had sexual relations with various people or having participated in group sex in the past. In one case the participant insinuated that this showed that the victim did not have credibility:

P4: I also have (pause) have witnesses from the people who helped me because they knew how she was. She would go with everybody (pause) from the school.

In other cases the participants suggested that it led them to believe that the victim would be a willing participant:

P9: But us, between ourselves (pause) because of the history that she already had (pause) of, of having relations with various.

This was also the case with one of the male victims who was a vulnerable young adult with a learning disability who had been taken advantage of in the past by other people:

P6: That guy there (pause) we did this, but I know people that also had (pause) or paid or something like that or they would buy him something.

### Influence of Alcohol and/or Drugs

Overall, almost one quarter (*n* = 6, 24.0%) of the participants mentioned the influence of alcohol and/or drugs. More than half (*n* = 4, 57.0%) of the adult participants stated that one of the main reasons that they participated in the assault was because they were either drunk or under the influence of drugs. This was a reason very rarely given by the juvenile participants and only two (11.0%) young participants (one who committed the offense with two adult co-offenders) said that it was a reason for being involved in the offense:

P6: I was at a party and so me and my friends had already drank a bit and then we got into some drugs and it was there that caused (pause) nothing else.

In some cases, the juvenile participants admitted to having drunk alcohol or smoked drugs but stated that it had not played a role in the offense:

P13: No, I don’t think so (pause) yes we had drunk (pause) but I think wine, but it was with 7Up (a fizzy drink), but many hours had passed since that happened.

P14: No, that happened not because because I smoked hash, which I always smoked since a child.

The adult participants who considered that alcohol had contributed to them being involved in the offense saw it as influencing their behavior and decisions:

P23: It was bad influence of the alcohol.

One of the participants was able to describe in more detail how that influence occurred and believed that it made him more susceptible to the influence of others:

P18: Then also with alcohol, I become, I become weak (pause) thinking is weaker. Oh so I go to show that I’m not a coward. That’s it, with drink with alcohol that is what I become. “You are a coward you won’t do this”. “Oh yeah, I won’t do it? Do you think that I won’t do it? Now I’m going to do it so that you can see”.

P18: And (pause) if it wasn’t for, if I wasn’t drunk I could have not gone because me with behavior of, with alcohol I’m one person, without alcohol another. With alcohol I don’t care about many things, without alcohol, but when I’m with alcohol I’m a person that goes. They pull me by the hand, say “Come,” say “Let us go walk for a while” I go. I’m like that decide (pause) decide easily.

### Normalized Sexual Violence

In a few of the juvenile cases, the participants referred to not being aware of the seriousness and consequences of their acts and a couple (8.0%) of participants mentioned how they had already witnessed similar situations in the past and that is why they did not think that it was serious. It is important to note that these participants came from poor, crime-prone neighborhoods where gang culture was common. One participant spoke quite extensively about how he had seen consensual group sex and multiple perpetrator rape occurring, and therefore, he thought that it was something normal:

P14: I got dressed and (pause) and then her friend appeared and said “Oh you brought her here for this! I thought it was to talk”. And I said “Oh you look like you don’t know, don’t know this (pause) this type of routine”. Routine but I say routine because (pause) I had already heard and seen some of these things, this type of thing and she knows, it had already happened to her but (pause) it was because she wanted to, not because she was forced, yes.

P14: No (pause) because I had already seen (pause) many episodes of those and (pause) and nothing happened and I said this isn’t more than something normal as well, as if I was stealing a mobile phone and that (pause) yes yeah.

P14: Sometimes they wanted to…they agreed and there were other days that I saw that they didn’t agree. I don’t want to say that it was always the same people, no, it was like normal, like I knew…yeah normal.

Another participant also spoke about situations of group sex that he had witnessed and stated that there was even a name for the type of girl that takes part in this activity:

P3: Don’t you know? (pause) Haven’t you ever heard that word “ger”?

Interviewer: What?

P3: “Ger”

Interviewer: No.

P3: It is a girl that goes to someone do you see? And the friend takes someone else and then both of them have relations with the girl do you understand?

Interviewer: So is it that frequent?

P3: Exactly but it is with consent because the girl lets.

## Discussion

This study examined the reasons that convicted perpetrators of MPSO gave for their involvement in an offense. Six main themes were identified which included: started as something else, influence of others (direct or indirect), lack of insight, victim blaming, influence of alcohol and/or drugs, and normalized sexual violence. However, in most cases the participants did not report just one main reason for being involved and usually described a combination of various factors and reasons. The results, therefore, support the existence of some of the factors proposed by the Multi-Factorial Model of MPSO (Harkins & Dixon, 2010, 2013) and earlier theories (see Table 3). The findings will be examined in more depth and discussed in the context of prior research in the area of general and sexual offending.

The theme *started as something else* (i.e., participants explained how they had not planned a sexual assault but that somehow it had happened) indicates that it is probable that a combination of individual, sociocultural and situational factors led to the assault. For example, in the situations where the participants said they were just having fun together, there may have been an interaction between individual traits (which could be related to personality, developmental factors, or sexual interests), beliefs about stereotypical masculinity, and a situation where co-offenders are present and are drunk, excited and/or aroused, as well as an available victim.

Research findings show that in the majority of group crimes there is little planning, and many occur, in part, due to impulsive behavior (Warr, 2002). Alarid, Burton, and Hochstetler (2009) found that street robbery committed by multiple perpetrators is often a spontaneous, impulsive opportunity that did not involve any planning. Furthermore, Matza (1964) suggested that young people can engage in criminal behavior without fully meaning to do so. Processes, such as behavioral contagion, can contribute to this unintended outcome (Polinsky, Lippitt, & Redl, 1950). Wheeler (1966) stated that behavioral contagion occurred when an individual performs an action and, as a result, another individual (who was uncertain whether or not to perform this action) acts in the same way. Contagion involves a circular process where members of a group do not examine the meaning of another member’s behavior (Blumer, 1951). This is facilitated by other processes such as deindividuation, which is discussed below.

Two individual characteristics in this sample which seem to be pertinent to this theme are the age and ethnicity of the offenders. The majority of the offenders were under 18 years old which is consistent with what is found in the MPSO literature (Amir, 1979; da Silva et al., 2014; Hauffe & Porter, 2009; Horvath & Kelly, 2009; Porter & Alison, 2006; Wright & West, 1981). Additionally, in the co-offending literature, young age has also been associated to co-offending (Reiss, 1988). Furthermore, it seems that there may be some differences between the young and the adult offenders as the adult offenders stated more frequently that alcohol played a part in their offense and that their co-offenders had a direct influence on their behavior. Etgar (2013) noted that in the literature on lone sexual offending, it has already been established that there are clear differences between adolescent and adult sexual offenders and that these should also be considered in order to tailor therapeutic interventions when working with perpetrators of MPSO. Etgar highlighted that these differences are apparent in emotions, cognitions, attitudes, and behaviors due to the fact that adolescents experience the world in a different way to adults. Furthermore, it is thought that adolescents’ sexually harmful behavior is rarely related to sexual deviance and is more often linked to their lack of perception regarding the harmful effects of their behavior (Ryan, 2010).

More than half of the offenders were from ethnic minority groups, which is also consistent with previous literature (Aebi, Vogt, Plattner, Steinhausen, & Bessler, 2012; Bijleveld & Hendriks, 2003; da Silva et al., 2014; De Wree, 2004; Horvath & Kelly, 2009; Woodhams, 2008). Past studies have shown that, especially in young incarcerated populations, ethnic minorities are over-represented (Bauer et al., 2011). Additionally, several risk factors for criminal behavior have been identified in ethnic minority groups which include discrimination in the host society, difficulties in acculturation and integration, and the socio-economic gap between ethnic minorities and nationals (Bauer et al., 2011; Mirsky, 2012).

Young age and ethnicity of offenders are not considered to be causal factors of MPSO, but these characteristics could be viewed as risk factors that, in association with other factors (e.g., sociocultural and situational), could increase the likelihood of engaging in this type of offending.

Clear evidence is provided for the existence of group processes and dynamics in some of the reasons given by the participants for being involved in MPSO. It is possible to identify group processes proposed by Harkins and Dixon (2013), such as social conformity and social comparison, in the theme related to the *influence of others*. Social comparison theory is related to an individual’s needs for affection and inclusion, whereby an individual may reluctantly go along with a sexual assault in an attempt to try to meet these needs (Harkins & Dixon, 2013). Social conformity reflects an individual striving to be consistent with the group norms by altering his beliefs, statements, or behaviors (Baron & Kerr, 2003). This conformity is influenced by rewards and punishments controlled by the group. Harkins and Dixon (2013) considered that some individuals would participate in MPSO to avoid being rejected or even punished by the group and losing rewards they received from the group. When the participants of the current study spoke about the influence of others, some of them directly stated that they did not want to look bad, to have problems with the group, or be rejected by their peers, which clearly points to the presence of social comparison and conformity. Others did not report these reasons directly, but disclosed obeying an order without questioning it, and others stated that they participated after the co-offenders either insisted they do or taunted them. This is suggestive of either being scared of the other co-offenders and not wanting to be punished by them, or wanting to belong to the group and therefore acting in a way that would demonstrate that they were part of it. These findings are consistent with previous studies which reported that the reasons that perpetrators of MPSO gave for participating in the assault were related to social comparison and conformity (Etgar & Ganot-Prager, 2009; Hooing et al., 2010).

Modeling is another group process that is relevant to the theme *influence of others*. O’Sullivan (1991) considered that this group process was relevant to MPSO because by watching peers sexually assault a victim, not only do the members of the group learn that it is acceptable, but also how to do it. In the current study, some participants reported how they took part after seeing their co-offenders assault the victim.

In the theme related to *lack of insight,* participants described not having insight into their feelings and thoughts and that the events happened quickly and in a confusing manner. This could indicate the influence of the group process deindividuation. Deindividuation is where a person loses his/her sense of individuality, becoming less self-conscious, and is submerged into the group (Goldstein, 2002). O’Sullivan (1991) believed that deindividuation could be responsible for a state of reduced self-awareness, including a reduced awareness of personal beliefs, attitudes, and standards. In the current study, some participants stated that they could not understand how they had assaulted the victim; that it was something that they had never thought about before. Harkins and Dixon (2013) considered that in MPSO, deindividuation could help to explain how a person can lose his/her sense of identity and responsibility and go along with the group.

In the theme *victim blaming,* sociocultural factors related to beliefs and attitudes about women, sexuality, rape myths, and gender norms were implicated. Some participants in the study spoke about how the female victim was judged by her past behavior. If she had, or was believed to have had, many sexual partners in the past, or to have participated in group sex, she was seen as someone who would be willing to have “sex” with all the group members. This was also apparent in a case with a male victim, who was a vulnerable young adult.

There were some distinct aspects to the cases involving male victims. In the majority of cases they were younger and physically weaker than the perpetrators. In three cases, they were school colleagues of the offenders and the offenses seem to have occurred in a bullying context. In one of the cases, the offenders admitted that their aim was to punish the victim because he had complained to the school when they had previously bullied him. In the only case in which the victim was an adult male, he had a learning disability and had in the past been abused by other people. A few authors have proposed that men targeted for MPSO are perceived by the perpetrators as not fitting into stereotypical gender norms because, for example, they are considered physically or mentally weak, or homosexual (Franklin, 2004; Lees, 2002).

Sociocultural factors can also be identified in the theme *normalized sexual violence*. More specifically, in this theme, sociocultural factors seem to be interacting with situational factors. Harkins and Dixon (2013) described this interaction as “subcultural context.” A couple of participants who came from crime-prone neighborhoods known for their gang culture explained how they considered what they had done to be normal because it was something that they had already witnessed and was acceptable in their circle of friends and acquaintances. This demonstrates how broader sociocultural factors (attitudes toward women and sexuality) can interact with situational factors (crime and gang culture) and increase the likelihood of MPSO.

Situational factors can be identified in the theme *influence of alcohol and/or drugs*. The participants that spoke about this theme considered that they would not have committed the assault if they were not under the influence of alcohol. They considered that the alcohol had a disinhibiting effect or had clouded their judgment. Nevertheless, they did not see it as the only factor and, in one of the quotes above, a participant explained how alcohol allowed him to become more susceptible to the influence of others. He felt that he had assaulted the victim not only because he was drunk, but because, by being drunk, he was more susceptible to the coercion and taunts of his co-offender. Studies have frequently found that in approximately half of sexual assaults, the perpetrator had been drinking alcohol (Abbey, Zawacki, Buck, Clinton, & McAuslan, 2004). Alcohol consumption can have a number of pharmacological and psychological effects on an individual (Abbey, 2011). It can hinder cognitive functions, such as reasoning, memory, and judgement, and impede response inhibition (Abroms, Fillimore, & Marczinsk, 2003). The alcohol myopia theory (Steele & Josephs, 1990) proposes that, when under the influence of alcohol, people tend to focus on the most immediate and salient cues. Therefore, they would not have the capacity to take in cues such as risks or future outcomes. There may also be an expectancy effect (Collins & Messerschmidt, 1993), whereby if a person believes that they will behave in a certain way when they drink alcohol, then they will behave in that way. It has been found that men expect to feel more aggressive and sexually aroused after drinking alcohol (Tuliao & McChargue, 2014). This is linked to the deviance disavowal theory (Miller, Maguin, & Downs, 1997), which suggests that some people use alcohol as an excuse for premeditated behaviors and then blame those behaviors on alcohol.

### Limitations

While self-reports from offenders make it possible to obtain their own accounts and opinions about their involvement in their offenses, they do have limitations. For example, some offenders may try to minimize or even deny their involvement in the offenses in order to present themselves in a more favorable light, which can affect the reliability of these accounts. Offenders often use minimizations and post hoc excuses when talking about their offenses. This is not surprising since, outside the criminal context, post hoc rationalizations and excuses are widely used by people when they do something that is perceived to be negative (Maruna & Mann, 2006). Excuse making has been described by Snyder and Higgins (1988) as an adaptive mechanism, which is important in maintaining self-esteem and coping with stress and anxiety. In addition, the post hoc explanations for their offending provided by the offenders might not be accurate because people can have little direct introspective access to their cognitive processes. Nisbett and Wilson (1977) suggested that when people try to explain the causes of their behavior they do so based on a priori implicit causal theories about the extent to which a certain stimulus is a believable cause of a given response.

In recognition of this, the offenders’ case files, which included detailed court accounts of the offenses, were read by the interviewer before the interviews and the offenders were informed of this.

In the main themes identified, there is very little reference to individual factors. During the interviews, a few offenders did speak about individual factors, such as going through a difficult period at the time the offense occurred because of family problems, or considering that at that time they were very young, immature, or irresponsible. Nevertheless, it was not a well-developed theme and this could be due to the fact that the focus of the interviews was on what happened directly before, during and after the assault, rather than specifically prompting for individual factors. This could be considered a limitation of this study and in future research it would be useful to explore possible individual factors.

Another limitation of this study is that the sample consisted exclusively of convicted offenders of MPSO. It is well known that a significant number of sexual assaults are not reported to the police (Walby & Allen, 2004). Furthermore, Andersson, Mhatre, Mqotsi, and Penderis (1998) found that victims of MPSO were less likely to report the crime to the police than victims of lone sexual violence. This makes it difficult to generalize the findings to unconvicted MPSO offenders, as the perpetrators’ experiences and explanations could be different. Further research using community samples is needed to overcome this limitation.

### Practical Implications

The results of this study have implications for prevention, assessment, and treatment purposes. Although a number of dynamic risk factors for sexual violence in general have been ascertained (e.g., Mann, Hanson, & Thornton, 2010), those specific to MPSO remain to be identified. For some of these offenders, MPSO-related factors might be the only dynamic risk factors present. The results from the current study highlight the importance of group processes in MPSO. These should, therefore, be identified and addressed in prevention and treatment programs.

Both Blanchard (1959) and Etgar and Ganot-Prager (2009) have noted previously that the dynamics observed between group members during evaluation and therapeutic intervention were similar to those reported to be present during the sexual assault itself. Therefore, as Etgar (2013) suggested, it is important to examine the perpetrator’s social role within the offending group. This will provide more information about the offender and his/her expected interactions in a group therapy setting, as well as possible risk factors which need addressing through intervention. For instance, if it is identified that an offender is susceptible to being influenced by others, therapeutic work could focus on increasing their self-control and assertiveness. A meta-analysis on the effectiveness of self-control programs among children and adolescents found that these interventions can reduce delinquency (Piquero, Jennings & Farrington, 2009). It has also been found that cognitive-behavioral treatment programs (e.g., the “Reasoning and Rehabilitation” program) that include components such as social skills training, assertiveness training, interpersonal training, and social perspective training are effective in reducing recidivism (Tong & Farrington, 2006). Similarly, with regard to prevention programs with young people, issues such as peer pressure and group processes should be addressed. Sullivan and Jolliffe (2012) evaluated a number of peer influence and mentoring programs which targeted peer relationships and decision making within the context of peer interactions, and found some promising results in relation to the reduction of delinquent behavior.

The findings support a multi-factorial explanation of MPSO which means that, besides group processes, other factors are also present and should be taken into account for prevention, assessment, and treatment purposes. Themes consistent with sociocultural and situational factors were identified that, in interaction with individual factors, likely led to the offense. Although more research is necessary to gain a better understanding of these factors and how they interact, Harkins and Dixon’s (2010, 2013) Multi-Factorial Model of MPSO provides a useful framework for understanding this type of sexual violence.

### Conclusions

As expected, group processes and dynamics were given as reasons for their involvement in MPSO by the offenders we interviewed. Additionally, other explanatory factors (i.e., sociocultural and situational) that had been proposed by Harkins and Dixon (2010, 2013), and in earlier theories (Amir, 1971; Brownmiller, 1975; Groth & Birnbaum, 1979; Sanday 2007) of MPSO, were present in the main themes identified from the interviews. Furthermore, the participants tended to attribute their offending to multiple factors, rather than just one. This supports the proposition of multiple, interacting factors explaining the perpetration of MPSO. These findings provide some evidence to support the Multi-Factorial Model of MPSO and other theories of MPSO that have been proposed (Amir, 1971; Groth & Birnbaum, 1979).

## Footnotes

1 The term multiple perpetrator rape was suggested by Horvath and Kelly (2009) as an overarching term for any sexual assault committed by two or more perpetrators and includes a broad range of sexual offenses. In this article, the term MPSO is used because it is the term utilized in the main theory examined.

2 For a more in depth description of this model and other earlier explanatory theories of MPSO, see da Silva, Woodhams, and Harkins (2015) and Harkins and Dixon (2010, 2013).
